# Tetrandrine Ameliorates Myocardial Ischemia Reperfusion Injury through miR-202-5p/TRPV2

**DOI:** 10.1155/2021/8870674

**Published:** 2021-03-08

**Authors:** Wei Zhao, Youyang Wu, Fanhao Ye, Shiwei Huang, Hao Chen, Rui Zhou, Wenbing Jiang

**Affiliations:** Department of Cardiology, The Third Clinical Institute Affiliated to Wenzhou Medical University, Wenzhou, 325000 Zhejiang, China

## Abstract

**Objective:**

This study is aimed at investigating the therapeutic effects of tetrandrine (Tet) on myocardial ischemia reperfusion (I/R) injury and probe into underlying molecular mechanism.

**Methods:**

H9C2 cells were divided into hypoxia/oxygenation (H/R) group, H/R+Tet group, H/R+Tet+negative control (NC) group, and H/R+Tet+miR-202-5p inhibitor group. RT-qPCR was utilized to monitor miR-202-5p and TRPV2 expression, and TRPV2 protein expression was detected via western blot and immunohistochemistry in H9C2 cells. Cardiomyocyte apoptosis was evaluated through detection of apoptosis-related markers and flow cytometry. Furthermore, myocardial enzyme levels were detected by ELISA. Rats were randomly separated into sham operation group, I/R group, I/R+Tet group (50 mg/kg), I/R+Tet+NC group, and I/R+Tet+miR-202-5p inhibitor group. miR-202-5p and TRPV2 mRNA expression was assessed by RT-qPCR. TRPV2 protein expression was detected through western blot and immunohistochemistry in myocardial tissues. Apoptotic levels were assessed via apoptosis-related proteins and TUNEL. Pathological changes were observed by H&E staining. Myocardial infarction size was examined by Evans blue-TCC staining.

**Results:**

Abnormally expressed miR-202-5p as well as TRPV2 was found in H/R H9C2 cells and myocardial tissues of I/R rats, which was ameliorated following Tet treatment. Tet treatment significantly suppressed H/R- or I/R-induced cardiomyocyte apoptosis. ELISA results showed that CK-MB and LDH levels were lowered by Tet treatment in H/R H9C2 cells and serum of I/R rats. H&E staining indicated that Tet reduced myocardial injury in I/R rats. Also, myocardial infarction size was lowered by Tet treatment. The treatment effects of Tet were altered following cotreatment with miR-202-5p inhibitor.

**Conclusion:**

Our findings revealed that Tet may ameliorate myocardial I/R damage via targeting the miR-202-5p/TRPV2 axis.

## 1. Introduction

Myocardial ischemia/reperfusion (I/R) is a process when the blood reperfusion caused by the recovery of blood flow after myocardial tissue ischemia, which could aggravate myocardial damage and dysfunction, thereby leading to myocardial infarction and even heart failure [1–3]. I/R injury can induce cell apoptosis and increase in infarct area. It has been a key factor that affects the treatment effect of acute myocardial infarction [4]. Therefore, it is of importance to explore novel treatment strategies to improve myocardial I/R damage.

MicroRNA (miRNA) is a membrane of the noncoding RNA family, with about 21 nucleotides in length [5]. miRNAs usually target multiple genes. Myocardial I/R injury involves various miRNAs [6]. It has been emphasized that miRNAs mediate the regulation of I/R-induced myocardial damage and dysfunction, which may be myocardial ischemic disease as a potential therapeutic target [7]. As a member of the miRNA family, miR-202-5p may participate in regulating the progression of various cancers. For example, it is related to breast cancer cell resistance to Adriamycin [8]. Furthermore, it may mediate biological behaviors of thyroid carcinoma cells [9]. Recently, the protective function of miR-202-5p against myocardial I/R damage has been reported via Transient Receptor Potential Cation Channel Subfamily V Member 2 (TRPV2) [10]. Ca^2+^ overload as well as mitochondrial dysfunction has been found to be stimulating factors for myocardial I/R injury [11]. TRPV2 is a family membrane of TRPV channel within and around the cardiovascular system [12]. Under pathological conditions, TRPV2 mediates abnormal Ca^2+^, thereby accelerating the progression of diseases [13]. It has been considered as a therapeutic target for cardiovascular diseases [14–16].

Tetrandrine (Tet) is a bisbenzylisoquinoline alkaloid extracted from *Stephania tetrandra* S. Moore plant [17]. Tet possesses a variety of pharmacological effects such as anticancer [18], anti-inflammation [19], and antioxidant stress [20]. Excessive apoptosis of cardiomyocytes is also the main cause of myocardial I/R damage [21]. Therefore, antiapoptosis therapy could reduce I/R injury and ameliorate cardiac dysfunction [22]. Cellular and animal experiments have demonstrated that Tet is effective for treatment of cardiovascular diseases [23]. However, it remains unclear concerning the effects and underlying molecular mechanism of Tet on myocardial I/R injury. Thus, a myocardial I/R injury model was constructed at the cellular and animal levels. We observed the therapeutic effect of Tet on myocardial I/R damage and explored its molecular mechanism. Our findings revealed that Tet could be a new therapeutic drug for myocardial I/R damage.

## 2. Materials and Methods

### 2.1. Cell Culture

H9C2 cells were grown in the Dulbecco's modified Eagle medium (SH30243.01B; Hyclone, Beijing, China) containing 10% fetal bovine serum (SH30084.03; Hyclone) at 37°C and 5% CO_2_ saturated humidity. These cells were separated into four groups: control group (without any treatment), hypoxia/oxygenation (H/R) group (hypoxia (120 min, 95% N_2_/5% CO_2_) and reoxygenation (30 min, 95% O_2_/5% CO_2_)), H/R+tetrandrine (Tet) group (H9C2 cells were exposed to H/R, followed by treatment with 1.5 *μ*mol/L Tet for 48 h), H/R+Tet+negative control (NC) group (H9C2 cells were exposed to H/R, treated with 1.5 *μ*mol/L Tet for 48 h, and transfected NC via Lipofectamine 2000 for 48 h), and H/R+Tet+miR-202-5p inhibitor group (H9C2 cells were exposed to H/R, treated with 1.5 *μ*mol/L Tet for 48 h, and transfected miR-202-5p inhibitor via Lipofectamine 2000 for 48 h).

### 2.2. Real-Time Quantitative PCR (RT-qPCR)

Takara minibest universal RNA extraction kit (9767; Takara) was utilized to extract total RNA from tissues or cells. 1 *μ*L RNA was measured at OD260 and OD280 with a UV spectrophotometer. According to the OD260/OD280 ratio, the RNA quality was estimated. Then, the total RNA was stored at -80°C. Then, reverse transcription was presented in the following reverse transcription system: 1 *μ*L PrimeScript enzyme mix, 1 *μ*L RT primer mix, 4 *μ*L 5x PrimeScript buffer, 3.1574 *μ*g RNA, and up to 20 *μ*L RNase-free H_2_O. Primer sequences were as follows: TRPV2: 5′-CGACGGGCTTCTACAAATGG-3′ (forward), 5′-AGGACCGTAACACCACTCAG-3′ (reverse); GAPDH: 5′-ACTCCCATTCTTCCACCTTTG-3′ (forward), 5′-CCCTGTTGCTGTAGCCATATT-3′ (reverse); miR-202-5p: 5′-TGCGCTTCCTATGCATATACT-3′ (forward), 5′-CAGTGCGTGTCGTGGAGT-3′ (reverse); and U6: 5′-GCTTCGGCAGCACATATACTAAAAT-3′ (forward), 5′-CGCTTCACGAATTTGCGTGTCAT-3′ (reverse). The reverse transcription reaction procedure was as follows: 37°C for 15 min, 85°C for 5 s and 4°C hold. RT-qPCR was utilized to quantify the mRNA expression of target genes by ABI 12K fluorescence RT-qPCR instrument (ABI, USA).

### 2.3. Cell Counting Kit-8 (CCK-8) Assay

Cell viability was determined utilizing CCK-8 detection kit (Dojindo, Shanghai, China). The cells in each group were seeded onto 96-well plates. Each well was treated with 10 *μ*L CCK-8 solution at 37°C for 3-4 h. After adding 10 *μ*L stop solution to each well, the OD450 value was determined with a microplate reader.

### 2.4. Flow Cytometry Assay

Cells were plated at 6-well plates (3∗10^5^ cells/well) overnight. After different treatments, the cells were harvested. Apoptotic levels were assessed by apoptosis detection kit (Vazyme, Nanjing, China). After centrifugation, 100 *μ*L Annexin Binding Buffer was used to resuspend the cells. The cells were, respectively, processed by 5 *μ*L Annexin FITC and 5 *μ*L PI at room temperature in the dark for 15 min. After adding 150 *μ*L Annexin Binding Buffer, apoptosis was detected using a flow cytometer (CytoFLEX S; Beckman, USA).

### 2.5. Western Blot

Tissues or cells were lysed by RIPA lysis buffer (P0013B; Beyotime, Beijing) on ice for 30 min. Following ultrasound in an ice bath for 3 min and centrifugation at 12000 rpm at 4°C for 10 min, the supernatant was transferred to a new EP tube. Using the BCA protein quantitative detection kit (P0009; Beyotime), the protein concentration was determined. The protein sample was separated through a polyacrylamide gel. After transferring the membrane, the sample was sealed in a 5% milk/TBST room at temperature for 1 h. The PVDF membrane was cocultured with the primary antibodies against TRPV2 (1 : 1000; BS-10297R; Bioss, Beijing), caspase3 (1 : 1000; AF7022; AFFINITY, Beijing), pro-caspase3 (1 : 3000; 19677-1-AP; Proteintech, Wuhan, China), Bax (1 : 3000; 60267-1-Ig; Proteintech), Bcl-2 (1 : 3000; 12789-1-AP; Proteintech), and GAPDH (60004-1-Ig; Proteintech) overnight at 4°C. Afterwards, the membrane was cocultured with the secondary antibody labeled with horseradish peroxidase (1 : 5000; SA00001-2; Proteintech) for 1 h at room temperature. The enhanced luminol reagent and oxidizing reagent were added to the membrane. After that, the membrane was colored by luminescent reagent 1.5 min. The results were observed with the gel imaging system.

### 2.6. ELISA

LDH and CK-MD levels were detected in cell culture fluid and serum samples by lactate dehydrogenase (LDH) detection kit (A020-2; Jiancheng, Nanjing, China) and creatine kinetic acid MD isoenzyme (CK-MB) detection kit (E0061-1; Jiancheng) in line with the manufacturer's instructions.

### 2.7. Animals

Totally, 25 male Sprague-Dawley rats with 220-250 g were purchased from Hangzhou Scientific Cloud Biotechnology Co., Ltd. (China). They were routinely housed in the Animal Experimental Center for 1 week. This study gained the approval of the Ethics Committee of The Third Clinical Institute Affiliated to Wenzhou Medical University (2018038). Experiments were presented in line with the recommendations in the Guide for the Care and Use of Laboratory Animals of the National Institutes of Health. All animals were randomly separated into five groups: sham operation (control) group, myocardial I/R group, I/R+Tet group (50 mg/kg), I/R+Tet+NC group, and I/R+Tet+miR-202-5p inhibitor group. After intraperitoneal injection of 1% sodium pentobarbital (40 mg/kg) to anesthetize the rats, the chest was opened and the left anterior descending (LAD) coronary artery was ligated for 30 min and then perfused for 120 min. The sternotomy was performed to expose the heart, followed by occlusion of the distal third of the coronary artery for 30 min through tightening the ligature. When cyanosis and protrusions occurred in the ischemic myocardium wall, the occurrence of ischemia was confirmed. The ligature was then removed, and the occluded coronary artery was reperfused for 120 min. In time of this period, the ECG confirmed that the ST segment recovered over 50%. Rats in the sham operation group received an open chest but did not ligate the coronary arteries. I/R rats were injected 50 mg/kg Tet through the tail vein after reperfusion. Rats in the sham operation group were injected with the same volume of saline. In the rats injected with Tet, miR-202-5p inhibitor or NC was injected through the tail vein 48 h until ligation of the LAD coronary artery. The rats were euthanized. Then, heart tissues and abdominal aorta blood were harvested. Tissues were fixed by 4% paraformaldehyde (E672002; Sangon Biotech, Shanghai, China).

### 2.8. Hematoxylin-Eosin (H&E) and Evans Blue-TCC Staining

After deparaffinization and hydration, the paraffin sections were separately stained by hematoxylin (Proteintech) as well as eosin (sigma, USA) for 3 min. Following mounting with neutral gum, images were taken under an optical microscope (OLYMPUS BX53; Olympus, Japan). Evans blue dye (Solarbio, Beijing, China) was injected into the aorta and coronary arteries to demarcate the ischemic risk or unstained areas of the heart. Following cutting the whole heart into five slices transversely, the slices were stained with 2% (*w*/*v*) triphenyltetrazolium chloride (TTC; T8877-25G; Sigma) at 37°C for 15 min. Image analysis software was used to quantify the infarct volume, risk area volume, and total heart volume. The percentage of infarct volume (%) was calculated by comparing to the whole heart.

### 2.9. Transferase-Mediated dUTP Nick End Labeling (TUNEL) Staining

Apoptotic levels were evaluated utilizing TUNEL apoptosis detection kit (ATK00001; AtaGenix, Wuhan). 10 U/mL DNase I and 1x DNase I Buffer were added in the positive control group, and 1x DNase I Buffer was added in the NC group. The sections were incubated with TUNEL detection solution in the dark for 60 min. Then, the sections were treated with 0.05 *μ*g/*μ*L DAPI solution for 10 min at room temperature and dark. Antifluorescence quenching mounting tablets were used for mounting. Pictures were taken under a fluorescence microscope (BX53; Olympus, Japan). By Image-Pro® Plus (IPP) software, 3 nonrepetitive and nonoverlapping visual fields were randomly chosen. The integrated optical density (IOD) values and areas for each field were determined. The average and standard deviation were calculated for statistical analysis.

### 2.10. Immunohistochemistry

Briefly, the sections were blocked with goat serum (C0265; Beyotime) at room temperature for 30 min. The sections were cotreated with primary antibody against TRPV2 (1 : 100; BS-10297R; Bioss) overnight at 4°C. Horseradish peroxidase-labeled anti-mouse IgG antibodies (ATPA00025Go; AtaGenix, Wuhan) were added to the sections and cultured at room temperature for 30 min. Then, the sections were colored by DAB for 5-10 min. Nuclei were counterstained with hematoxylin (B600020; Proteintech) for 3-5 min. After dehydration and transparency, the sections were sealed with neutral gum.

### 2.11. Statistical Analysis

All statistical analysis was presented through GraphPad Prism 7.0. Data were presented as the mean ± standard deviation. The differences between ≥3 groups were compared via one-way analysis of variance. *p* value < 0.05 was considered statistically significant.

## 3. Results

### 3.1. Tet Ameliorates the miR-202-5p/TRPV2 Axis in H/R-Treated H9C2 Cells

**Figure 1:**
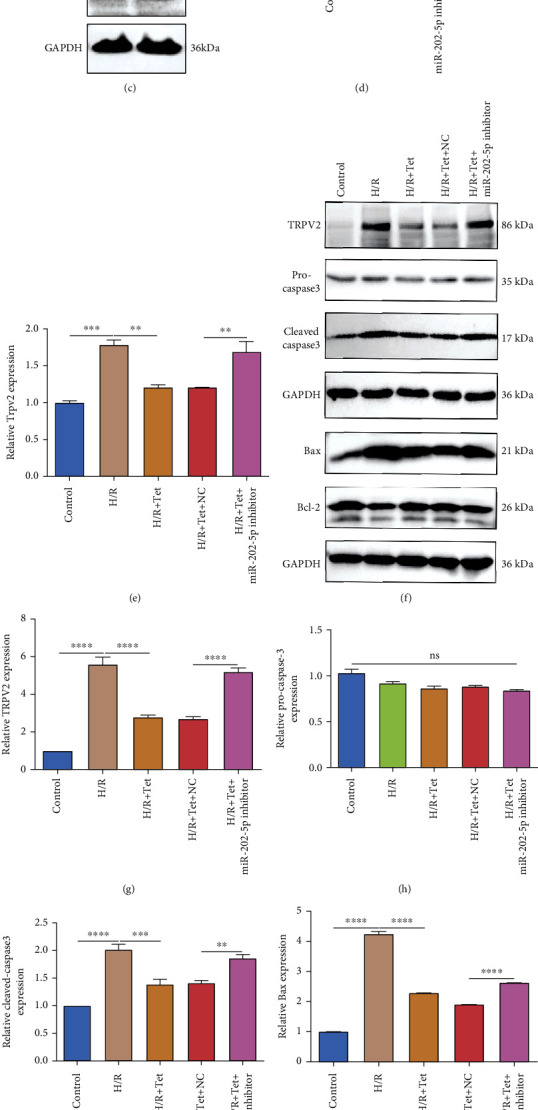
Tet ameliorates the miR-202-5p/TRPV2 axis in H/R H9C2 cells. (a) RT-qPCR detecting the expression of miR-202-5p in H/R H9C2 cells treated with Tet and/or miR-202-5p inhibitor. (b) The transfection of miR-202-5p inhibitor was evaluated in H9C2 cells by RT-qPCR. (c, d) Western blot showing the protein expression of TRPV2 in H9C2 cells transfected with miR-202-5p inhibitor. (e) RT-qPCR detecting the mRNA expression of TRPV2 in H/R H9C2 cells treated with Tet and/or miR-202-5p inhibitor. (f–k) Western blot showing the protein expression of TRPV2, pro-caspase3, cleaved caspase3, Bax, and Bcl-2 in H/R H9C2 cells treated with Tet and/or miR-202-5p inhibitor. ns: not significant; ^∗∗^*p* < 0.01; ^∗∗∗^*p* < 0.001; ^∗∗∗∗^*p* < 0.0001.

In the H/R-treated H9C2 cells, miR-202-5p expression was detected to be decreased in comparison to controls (Figure 1(a)), consistent with a previous study [10]. Tet treatment distinctly ameliorated H/R-induced suppression of miR-202-5p level, which was reversed after cotransfection with miR-202-5p inhibitor. The transfection effects of miR-202-5p inhibitor were assessed in H9C2 cells by RT-qPCR. The data confirmed that miR-202-5p expression was distinctly suppressed after transfection of its inhibitor compared controls (Figure 1(b)). As a previous study, TRPV2 has been identified as a direct target of miR-202-5p in rat cardiomyocytes [10]. Herein, we also found that the expression of TRPV2 protein was significantly lowered following transfection with miR-202-5p inhibitor in H9C2 cells (Figures 1(c) and 1(d)). In Figure 1(e), TRPV2 mRNA had a higher expression in H/R-induced H9C2 cells than controls. Its mRNA expression was suppressed in H/R H9C2 cells after treatment with Tet. However, miR-202-5p inhibitor could reverse the decrease in TRPV2 mRNA expression induced by Tet in H/R H9C2 cells. Furthermore, we examined the expression of TRPV2 protein through western blot. Similar to the RT-qPCR results, TRPV2 protein exhibited a distinctly higher expression in H/R H9C2 cells compared with controls (Figures 1(f) and 1(g)). Following treatment with Tet, its expression was prominently lowered in H/R-induced H9C2 cells, which was elevated through cotransfection with miR-202-5p inhibitor. Thus, Tet treatment could ameliorate the miR-202-5p/TRPV2 axis in H/R-induced H9C2 cells.

### 3.2. Tet Treatment Suppresses H/R-Induced Apoptosis in H9C2 Cells through miR-202-5p

**Figure 2:**
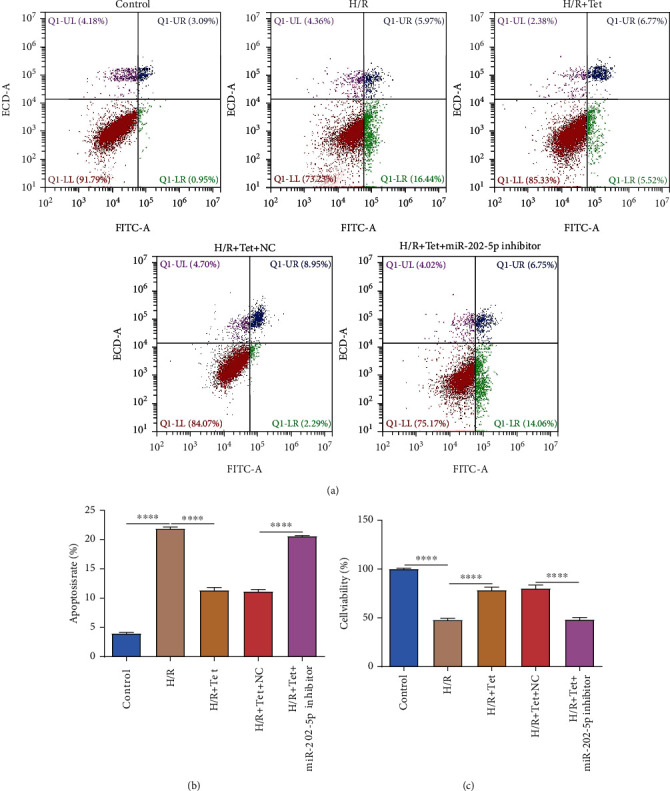
Tet treatment inhibits H/R-induced cardiomyocyte apoptosis through miR-202-5p. (a) Representative images of flow cytometry results. (b) Quantitative results of apoptosis levels for H/R H9C2 cells treated with Tet and/or miR-202-5p inhibitor. (c) CCK-8 assay was presented to assess the cell viability of H/R H9C2 cells treated with Tet and/or miR-202-5p inhibitor. ^∗∗∗∗^*p* < 0.0001.

We further observed whether Tet treatment could ameliorate H/R-induced apoptosis in H9C2 cells through miR-202-5p. The expression of apoptosis-related markers including pro-caspapse3, cleaved caspase3, Bax, and Bcl-2 was examined by western blot. There was no significant difference in pro-caspapse3 expression in these groups (Figures 1(f) and 1(h)). The expression of cleaved caspase3 (Figures 1(f) and 1(i)) and Bax (Figures 1(f) and 1(j)) was both markedly elevated in H/R H9C2 cells, which was improved by Tet treatment. With cotreatment with Tet and miR-202-5p inhibitor, cleaved caspase3 and Bax expression was distinctly higher in H/R H9C2 cells in comparison to those with Tet treatment. In Figures 1(f) and 1(k), Bcl-2 expression was significantly decreased in H/R H9C2 cells than controls, which was ameliorated by Tet treatment. However, miR-202-5p inhibitor weakened the treatment effects of Tet on Bcl-2 expression in H/R H9C2 cells. Apoptotic levels of H9C2 cells were also evaluated via flow cytometry. Apoptosis levels of H/R cells were significantly increased in comparison to controls (Figures 2(a) and 2(b)). Tet treatment could ameliorate H/R-induced apoptosis in H9C2 cells, which was reversed after cotreatment with miR-202-5p inhibitor. As depicted in CCK-8 assay results, the cell viability was significantly lowered in H/R-induced H9C2 cells than controls (Figure 2(c)). Tet treatment improved proliferation of H/R cells. Nevertheless, following cotreatment with miR-202-5p inhibitor and Tet, the proliferative capacity of H/R cells was distinctly decreased (Figure 2(c)). Thus, Tet treatment could suppress H/R-induced cardiomyocyte apoptosis, which could be partly related to miR-202-5p.

### 3.3. Tet Ameliorates the miR-202-5p/TRPV2 Pathway in Myocardial Tissues of I/R Rats

**Figure 3:**
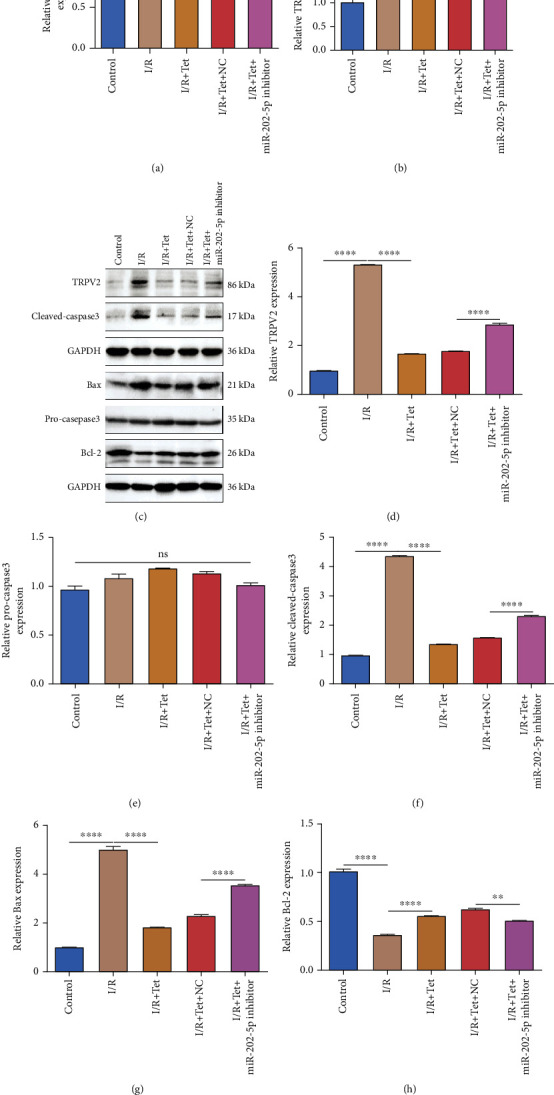
Tet ameliorates the miR-202-5p/TRPV2 pathway in myocardial tissues of I/R rats. (a, b) RT-qPCR examining the expression of miR-202-5p as well as TRPV2 in I/R rats treated with Tet and/or miR-202-5p inhibitor. (c–h) Western blot detecting the protein expression of TRPV2, pro-caspase3, cleaved caspase3, Bax, and Bcl-2 in I/R rats treated with Tet and/or miR-202-5p inhibitor. ^∗^*p* < 0.05; ^∗∗^*p* < 0.01; ^∗∗∗∗^*p* < 0.0001.

RT-qPCR results exhibited that miR-202-5p had a lower expression in myocardial tissues of I/R rats in comparison to sham operation (Figure 3(a)). After treatment with 50 mg/kg Tet, its expression was distinctly elevated in myocardial tissues of I/R rats. However, following cotreatment with Tet and miR-202-5p inhibitor, its expression was lowered in I/R rats. In Figure 3(b), TRPV2 mRNA had a higher expression in I/R myocardial tissues than controls. Following treatment with Tet, its mRNA expression was reduced in I/R rats. However, during cotreatment with Tet and miR-202-5p inhibitor, TRPV2 mRNA expression was elevated in myocardial tissues of I/R rats compared to those treated with Tet (Figure 3(b)). Western blot was also presented to examine TRPV2 expression in myocardial tissues. As shown in Figures 3(c) and 3(d), higher expression of TRPV2 protein was detected in I/R myocardial tissues than controls. Tet treatment prominently decreased its expression in I/R myocardial tissues, which was reversed following cotreatment with miR-202-5p inhibitor. Apoptosis-related proteins were also detected by western blot. No significant difference in pro-caspase3 expression was found in myocardial tissues in different groups (Figures 3(c) and 3(e)). The expression of cleaved caspase3 (Figures 3(c) and 3(f)) and Bax (Figures 3(c) and 3(g)) was elevated in I/R myocardial tissues than controls. After treatment with Tet, their expression was significantly decreased. However, inhibition of miR-202-5p distinctly augmented cleaved caspase3 and Bax expression in myocardial tissues of I/R rats treated with Tet. Moreover, Bcl-2 expression was distinctly lowered in myocardial tissues of I/R rats than controls, which was improved by Tet treatment (Figures 3(c) and 3(h)). However, miR-202-5p inhibitor weakened the effects of Tet on Bcl-2 expression in myocardial tissues of I/R rats. Taken together, Tet treatment improved the miR-202-5p/TRPV2 axis in I/R-induced myocardial damage.

### 3.4. Tet Decreases TRPV2 Expression in I/R Myocardial Tissues via Increasing miR-202-5p

**Figure 4:**
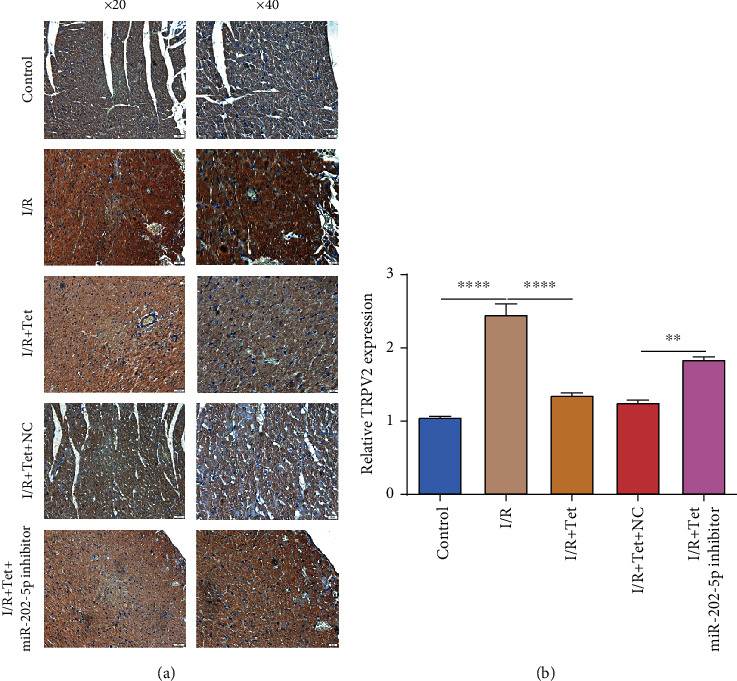
Tet decreases TRPV2 expression in I/R rats via increasing miR-202-5p. (a) Representative images of immunohistochemistry results for TRPV2 protein in I/R myocardial tissues treated with Tet and/or miR-202-5p inhibitor. Scar bar: 50 and 20 *μ*m. Magnification: ×20 and ×40. (b) The expression of TRPV2 protein was quantified in myocardial tissues of each group. ^∗∗^*p* < 0.01; ^∗∗∗∗^*p* < 0.0001.

Using immunohistochemistry, we examined TRPV2 expression in myocardial tissues of each group. As shown in Figures 4(a) and 4(b), TRPV2 protein had a significantly higher expression in I/R myocardial tissues compared to controls. Following treatment with Tet, its expression was distinctly suppressed in I/R myocardial tissues. However, cotreatment of miR-202-5p inhibitor altered the decrease in TRPV2 expression induced by Tet in I/R myocardial tissues. Therefore, Tet could suppress TRPV2 expression in I/R myocardial tissues, partly related to miR-202-5p activation.

### 3.5. Tet Lowers Levels of Cardiac Marker Enzymes in H/R Cardiomyocytes and Serum of I/R Rats via miR-202-5p

**Figure 5:**
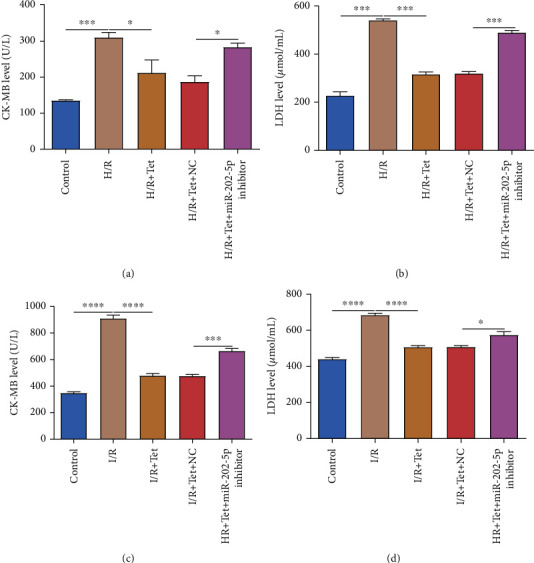
Tet lowers levels of cardiac marker enzymes in H/R-treated H9C2 cells and I/R rats via miR-202-5p. (a, b) ELISA results showing the levels of CK-MB and LDH in H/R H9C2 cells treated with Tet and/or miR-202-5p inhibitor. (c, d) ELISA detecting CK-MB and LDH levels in serum of I/R rats following treatment with Tet and/or miR-202-5p inhibitor. ^∗^*p* < 0.05; ^∗∗∗^*p* < 0.001; ^∗∗∗∗^*p* < 0.0001.

ELISA was carried out to detect the levels of myocardial enzymes including CK-MB and LDH in H/R H9C2 cells and serum of I/R rats. In Figures 5(a) and 5(b), CK-MB and LDH levels were prominently gained in H/R-treated H9C2 cells. Tet could decrease their levels in H/R H9C2 cells. However, after inhibiting miR-202-5p, their levels were distinctly augmented in H/R cardiomyocytes treated with Tet (Figures 5(a) and 5(b)). Furthermore, in serum of I/R rats, CK-MB and LDH levels were both significantly higher than controls (Figures 5(c) and 5(d)). Tet treatment markedly decreased their levels in serum of I/R rats. But miR-202-5p inhibitor reversed their high levels in I/R rats treated with Tet. Thus, Tet may lower levels of cardiac marker enzymes in H/R cardiomyocytes as well as serum of I/R rats partly via activation of miR-202-5p.

### 3.6. Tet Improves I/R-Induced Myocardial Infarction Size via miR-202-5p

**Figure 6:**
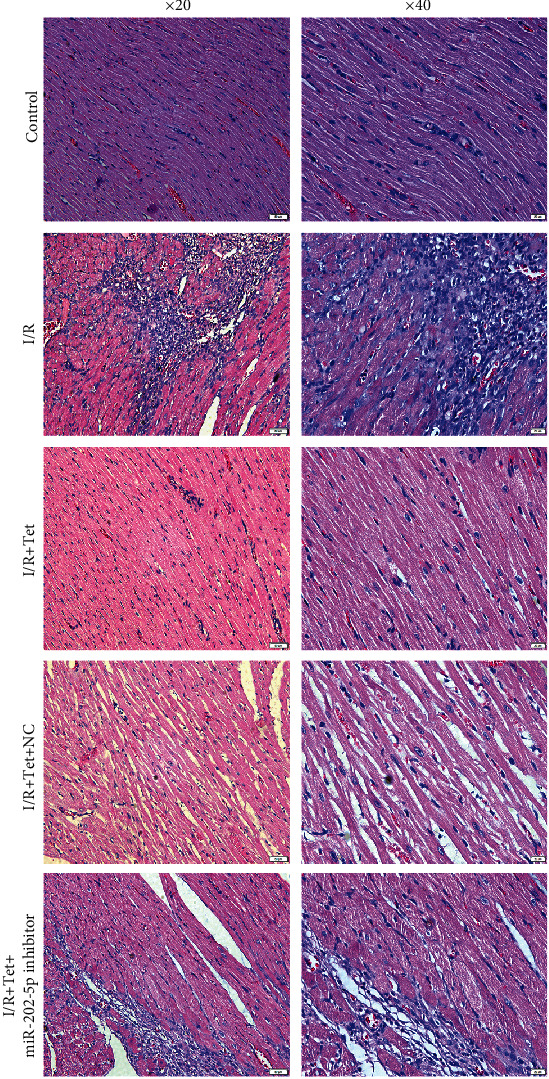
H&E staining showing the pathological changes in I/R myocardial tissues after treatment with Tet and/or miR-202-5p inhibitor. Scar bar: 50 and 20 *μ*m. Magnification: ×20 and ×40.

**Figure 7:**
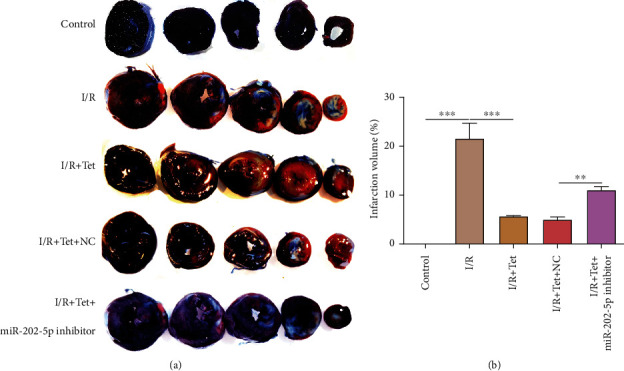
Evans blue-TCC staining detecting the myocardial infarction size in I/R rats treated with Tet and/or miR-202-5p inhibitor. (a) Representative images of Evans blue-TCC staining results. (b) The myocardial infarction size was quantified in each group. ^∗∗^*p* < 0.01; ^∗∗∗^*p* < 0.001; ^∗∗∗∗^*p* < 0.0001.

H&E staining was used to investigate the myocardial damage in each group. As shown in Figure 6, myocardial injury was obvious in I/R-induced rats, which was distinctly ameliorated following treatment with Tet. However, myocardial injury was observed for I/R rats treated with Tet and miR-202-5p inhibitor. Myocardial infarction size was monitored via Evans blue-TCC staining. In Figures 7(a) and 7(b), myocardial infarction was obvious in I/R rats, improved by Tet treatment. miR-202-5p inhibitor could increase myocardial infarction volume in I/R rats treated with Tet.

### 3.7. Tet Decreases I/R-Induced Myocardial Apoptosis via miR-202-5p

**Figure 8:**
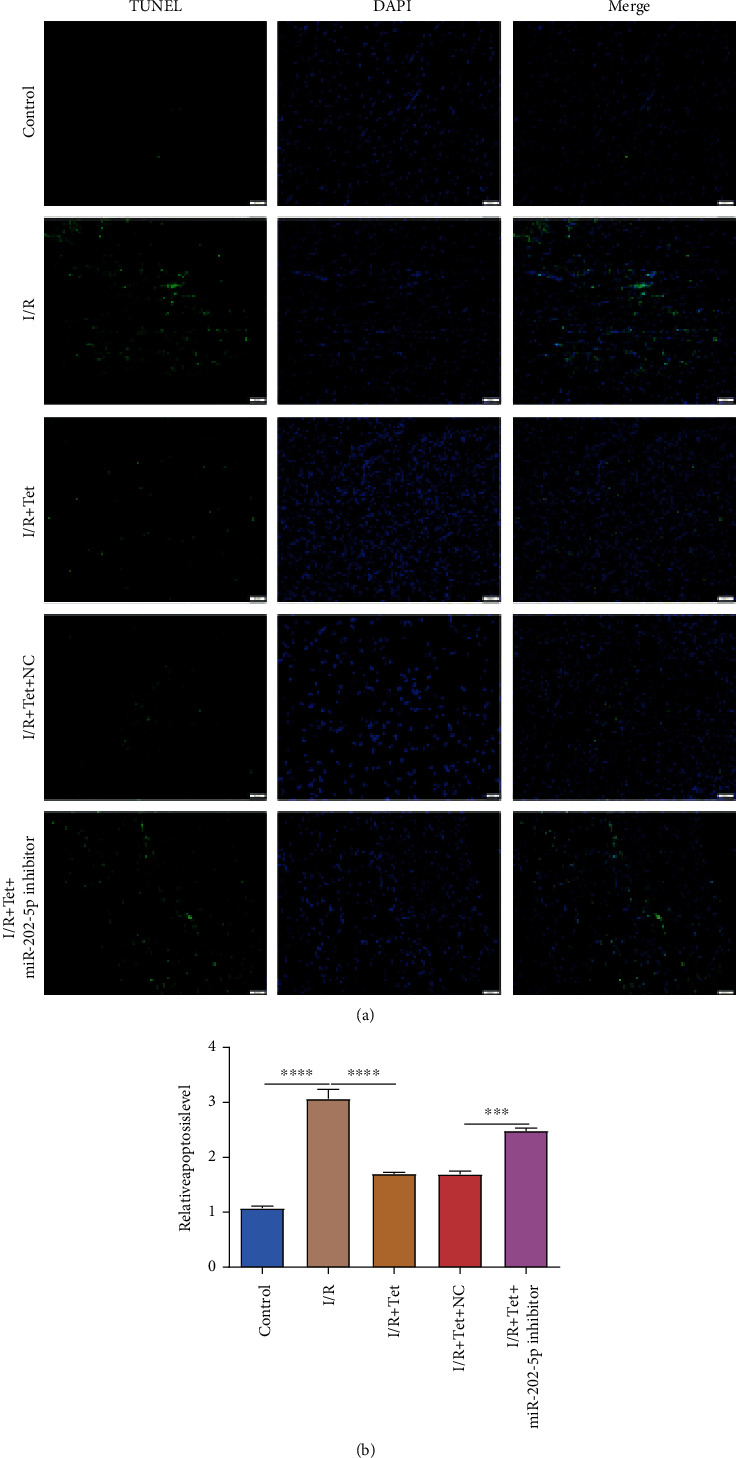
Tet reduces myocardial apoptosis in I/R rats via miR-202-5p. (a) Representative images of TUNEL staining results. Scar bar: 50 *μ*m. (b) Cardiomyocyte apoptosis was quantified in I/R rats treated with Tet and/or miR-202-5p inhibitor. ^∗∗∗^*p* < 0.001; ^∗∗∗∗^*p* < 0.0001.

Apoptosis in cardiomyocytes was examined via TUNEL staining. Our data demonstrated that cardiomyocyte apoptosis was markedly induced in I/R rats (Figures 8(a) and 8(b)). Following treatment with Tet, cardiomyocyte apoptosis was significantly ameliorated in I/R rats. miR-202-5p inhibitor improved cardiomyocyte apoptotic levels in I/R rats treated with Tet. Therefore, Tet treatment could reduce I/R-induced myocardial apoptosis partly via miR-202-5p.

## 4. Discussion

In this study, Tet could ameliorate cardiomyocyte apoptosis both in H/R cardiomyocytes cells and I/R rats. After treatment, I/R-induced myocardial infarction size was distinctly decreased. Previous studies have confirmed the protective roles of miR-202-5p on H/R cardiomyocytes and I/R myocardial tissues [10]. Hence, in this study, we investigated whether Tet treatment could increase miR-202-5p expression, thereby exerting the therapeutic effects on myocardial I/R damage. As expected, Tet increased miR-202-5p expression and decreased TRPV2 that was a target of miR-202-5p in myocardial I/R injury models. However, inhibiting miR-202-5p weakened the treatment effects of Tet. Thus, Tet could protect against myocardial I/R damage, which could have a close relationship with the miR-202-5p/TRPV2 axis.

miRNAs play a considerable part in regulating I/R-induced myocardial damage, which may be potential therapeutic targets for myocardial ischemic diseases [24–26]. Consistent with a previous study, miR-202-5p expression is lowered in H/R cardiomyocytes as well as I/R myocardial tissues [10]. Overexpression of miR-202-5p could ameliorate damage in H/R cardiomyocytes and I/R myocardial tissues. It has been found that miR-202-5p is involved in the regulation of cardiomyocyte autophagy [27]. Combining previous studies, inhibiting miR-202-5p may promote myocardial I/R injury. Thus, in this study, there were no miR-202-5p inhibitor and H/R+miR-202-5p groups. Herein, Tet treatment distinctly increased miR-202-5p expression in H/R cardiomyocytes as well as I/R myocardial tissues. Confirmed by dual luciferase report, miR-202-5p could directly bind to the 3′UTR region of TRPV2 [10]. TRPV2, as a target of miR-202-5p, is highly expressed in acute myocardial infarction tissues, consistent with our study results [28]. Overexpression of miR-202-5p could reduce myocardial I/R injury by inhibiting TRPV2. Studies have found that inhibiting TRPV2 activity can effectively improve heart function in patients with heart failure [29]. Tet treatment decreased miR-202-5p expression in H/R cardiomyocytes as well as I/R myocardial tissues. Thus, Tet ameliorated myocardial I/R injury, which may have a relationship with miR-202-5p and TRPV2.

Myocardial I/R damage exhibits a distinct relationship with cell apoptosis. Inhibition of apoptosis is a promising treatment strategy for I/R damage [30]. Our results showed that Tet treatment distinctly suppressed cardiomyocyte apoptosis in H/R-induced cardiomyocytes via flow cytometry. Moreover, according to TUNEL results, cardiomyocyte apoptosis was markedly ameliorated by Tet treatment in I/R myocardial tissues. Both in vitro and in vivo, cleaved caspase3 and Bax expression was suppressed and Bcl-2 expression was activated by Tet treatment. Previously, Tet could induce apoptosis of different cancer cells. For example, Tet can induce apoptosis of bladder cancer cells via the AMPK/mTOR axis [31]. Apoptosis of lung cancer cells is suppressed by Tet treatment through the VEGF/HIF-1*α*/ICAM-1 pathway [32]. Based on these studies, Tet could be a promising drug for improvement of cardiomyocyte apoptosis in myocardial I/R damage.

It has been verified that Tet can improve cardiac function and weaken the progression of cardiac hypertrophy [33]. In H/R-induced cardiomyocytes as well as serum of I/R rats, Tet treatment significantly decreased the levels of myocardial enzymes (CK-MB and LDH), indicating the myocardial protection function was significantly ameliorated by Tet treatment. H&E depicted that I/R-induced myocardial damage was markedly reduced following treatment with Tet. More importantly, myocardial infarction was prominently lessened for I/R rats treated by Tet. Our results revealed that Tet was a promising drug for treatment of myocardial I/R damage. Tet elevated miR-202-5p expression both in H/R cardiomyocytes and I/R myocardial tissues. After inhibiting miR-202-5p, the treatment effect of Tet on myocardial I/R injury was weakened. Thus, Tet exhibited a protective function against myocardial I/R damage, which could be in relationship with the miR-202-5p/TRPV2 axis.

## 5. Conclusion

Taken together, both in H/R cardiomyocytes and I/R rats, Tet may reduce myocardial I/R damage. Mechanically, Tet activated the miR-202-5p/TRPV2 pathway, thereby ameliorating myocardial I/R damage. Thus, Tet could become an underlying novel drug for treating myocardial I/R damage.

## Data Availability

The datasets analyzed during the current study are available from the corresponding author on reasonable request.

## Abbreviations

Tet: Tetrandrine
H/R: Hypoxia/oxygenation
NC: Negative control
I/R: Ischemia reperfusion
miRNAs: MicroRNAs
RT-qPCR: Real-time quantitative PCR
CCK-8: Cell Counting Kit-8 assay
LDH: Lactate dehydrogenase
CK-MB: Creatine kinetic acid MD
H&E: Hematoxylin-eosin
TUNEL: Transferase-mediated dUTP nick end labeling.
